# D-Loop Mutations as Prognostic Markers in Glioblastoma—A Pilot Study

**DOI:** 10.3390/ijms25084334

**Published:** 2024-04-14

**Authors:** Bartosz Szmyd, Patrycja Stanisławska, Małgorzata Podstawka, Karol Zaczkowski, Patryk M. Izbiński, Dominika Kulczycka-Wojdala, Robert Stawski, Karol Wiśniewski, Karolina Janczar, Marcin Braun, Piotr Białasiewicz, Dariusz J. Jaskólski, Ernest J. Bobeff

**Affiliations:** 1 Department of Neurosurgery and Neuro-Oncology, Barlicki University Hospital, Medical University of Lodz, 90-153 Lodz, Poland; bartosz.szmyd@umed.lodz.pl (B.S.); patrycja.stanislawska@stud.umed.lodz.pl (P.S.); podstawkagosia@gmail.com (M.P.); karol.zaczkowski@stud.umed.lodz.pl (K.Z.); patryk.izbinski@barlicki.pl (P.M.I.); karol.wns@gmail.com (K.W.); dariusz.jaskolski@umed.lodz.pl (D.J.J.); 2 Central Scientific Laboratory, Medical University of Lodz, 93-513 Lodz, Poland; dominika.kulczycka-wojdala@umed.lodz.pl; 3 Department of Clinical Physiology, Medical University of Lodz, 92-215 Lodz, Poland; robert.stawski@umed.lodz.pl; 4 Department of Pathology, Medical University of Lodz, 92-213 Lodz, Poland; karolinajanczar@googlemail.com (K.J.); marcin.braun@umed.lodz.pl (M.B.); 5 Department of Sleep Medicine and Metabolic Disorders, Medical University of Lodz, 92-215 Lodz, Poland; piotr.bialasiewicz@umed.lodz.pl

**Keywords:** glioblastoma, mitochondrial DNA, D-loop variants, D-loop m.16126T>C, prognosis, biomarkers

## Abstract

Glioblastoma, a highly aggressive brain tumor, poses significant treatment challenges. A deeper investigation into its molecular complexity is essential for the identification of novel prognostic biomarkers and therapeutic strategies, potentially improving patient outcomes in terms of survival and quality of life. While nuclear DNA mutations have been extensively studied, the role of mitochondrial DNA (mtDNA) mutations, specifically in the D-loop region, remains poorly understood. This prospective case-control study aimed to assess the prognostic significance of the mtDNA D-loop m.16126T>C variant in glioblastoma patients. Immunohistochemistry and droplet digital PCR (ddPCR) were employed for mutation analysis, complemented by statistical analyses and a literature review. The study cohort comprised 22 glioblastoma patients (mean age 59.36 ± 14.17, 12 (54.55%) females), and 25 controls (59.48 ± 13.22, 12 (80%) females). The D-loop m.16126T>C variant was observed in four (18%) of the glioblastoma samples and was associated with shorter median survival (9.5 vs. 18 months; *p* = 0.016, log-rank test). This study underscores the importance of investigating mtDNA, especially D-loop variants, in glioblastoma, suggesting its potential as a prognostic biomarker and, therefore, its possible therapeutic targets, warranting further exploration.

## 1. Introduction

Following the introduction of the fourth (2016) penultimate version of the WHO Classification of Tumors of the Central Nervous System, there has been a notable emphasis within the neuropathologists’ and neurooncologists’ community on the importance of establishing criteria based on the findings of tumor molecular biology [1]. This paradigm shift in neuropathological thinking has not only prompted the creation of a new fifth classification but catalyzed an increase in studies investigating molecular diagnostic and prognostic markers of brain tumors.

Despite the introduction of 22 new tumor entities and the revision of the terminology of 13 tumors, glioblastoma remains one of the most malignant and the most common primary intra-axial brain tumors [2]. And, therefore, many scientific efforts are focused on molecular biology to find new biomarkers and explore potential new treatment options. Glioblastoma is characterized by rapid cell proliferation and an infiltrative spread along white matter fibers. Despite specialized treatment, the 2-year survival rate does not exceed 18% [3]. Given the lack of effective treatment methods, understanding the role of genetic mutations in the development, progression, and drug resistance of this tumor is crucial.

Genetic alterations play a key role in glioblastoma progression, offering insight into therapeutic responses and prognosis. Initial attention was given to chromosomal abnormalities, recognized as potent biomarkers affecting treatment outcomes and survival prognosis [4]. Later studies revealed key mutations, such as those associated with *EGFR*, *IDH1/2*, *TP53*, and *PTEN*, shedding light on dysregulated pathways driving tumor proliferation and invasion [5]. However, the role of mitochondrial DNA in glioma progression remains to be fully elucidated.

Mitochondria participate in cellular respiration, the generation of free radicals, and the regulation of cell sensitivity to oxidative stress. As independent organelles, they contain their genome with a specific number of copies, known as the mitochondrial genome (mtDNA). Mutations in mtDNA disrupt cell metabolism, which can lead to tumor formation, increase tumor tolerance to hypoxia, and enhance free radical production [6]. They likely reduce the effectiveness of radiotherapy and induce chemotherapy resistance [7,8].

MtDNA replication initiates at the D-loop region where two DNA strands are separated and held apart by a third displaced DNA strand. In electron micrographs, it forms a loop resembling the “D” shape [9]. The D-loop participates in repairing DNA double-strand breaks (DSB) through homologous recombination (HR). During this process, the damaged DNA strand is matched to an undamaged DNA strand, creating a D-loop and displacing the other undamaged DNA strand. HR is essential for DSB repair during both meiotic recombination and mitotic division [10]. However, the specific involvement of the D-loop in glioblastoma development remains incompletely understood [11,12,13,14,15,16,17,18,19,20,21]. For the purpose of the current study, we decided to choose the D-loop m.16126T>C variant located in the hypervariable region (HV-I), which is characterized by a 100- to 200-fold higher mutation rate than nuclear DNA [22]. This mutation was, further, linked with poor event-free survival among pediatric patients with acute myeloid leukemia [22].

Increasing reports demonstrate the efficacy of liquid biopsy techniques employing circulating tumor DNA, circulating tumor cells, and extracellular vesicles for enhancing and expediting treatment [23]. In our recent study, we confirmed the diagnostic value of serum amino acids in glioblastoma patients [24]. There are several clinical applications such as establishing a diagnosis in the absence of tumor fragments, monitoring the treatment response, distinguishing progression from pseudoprogression, and predicting the treatment outcome.

We hypothesized that mtDNA mutations in the D-loop region have prognostic value and could contribute to the development of personalized therapy in glioblastoma.

## 2. Results and Discussion

### 2.1. Results

The study included 22 patients in the study group and 25 patients in the control group. The demographic and clinical characteristics of both groups are summarized in Table 1. In the study group, the mean age was 59.36 ± 14.17, while in the control group, it was 59.48 ± 13.22. The distribution of sex was 12 females and 10 males in the study group, and 12 females and 13 males in the control group. All patients received radiotherapy and chemotherapy according to the Stupp protocol.

#### 2.1.1. Molecular Marker Analysis

Tumor mtDNA D-loop m.16126T>C mutations were observed in four (18%) of the glioblastoma samples, while no mutations were detected in the plasma samples.

GFAP expression was detected in all tumors. The median Ki67 labeling index was 30% (IQR: 19–40). ATRX loss was observed in 11 (50%) cases, and P53 expression was noted in 6 (27%) cases.

#### 2.1.2. Clinical Outcome

The 12-month mortality rate was 41% (n = 9), and the overall mortality during the follow-up period was 55% (n = 12). The median survival time was 9 months (IQR: 6–14), and the median follow-up duration was 14 months (IQR: 9–16). At the time of publication, ten patients in the study group remained on post-radiotherapy temozolomide treatment. No data on clinical outcomes were available for the control group.

#### 2.1.3. Survival Analysis

**Figure 1:**
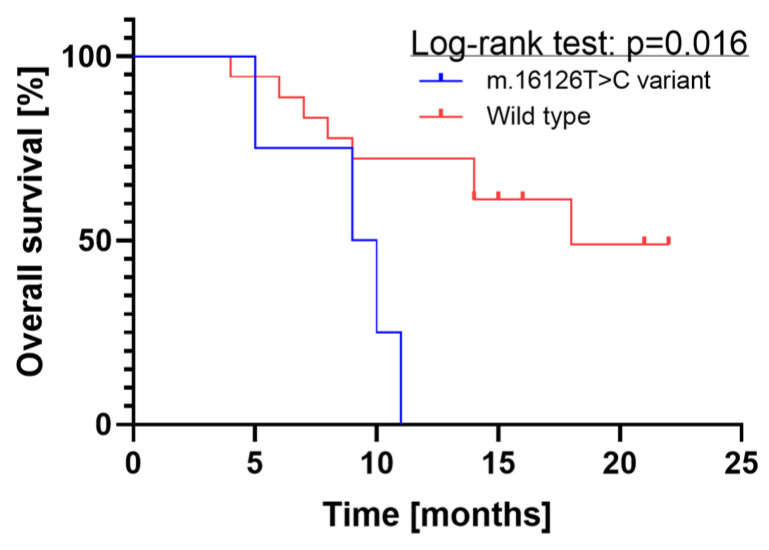
Kaplan–Meier plot depicting survival probabilities of glioblastoma patients based on mtDNA D-loop m.16126T>C mutation status. The *x*-axis represents the follow-up duration in months, while the *y*-axis indicates the probability of survival. The plot showcases distinct survival curves for patients with D-loop mutations versus wild-type D-loop, highlighting differences in survival outcomes between the two groups.

The log-rank test results revealed a significant difference in overall survival between the two groups (*p* = 0.016), as illustrated in Figure 1, which displays the Kaplan–Meier plot. There were four glioblastoma patients in the mtDNA D-loop m.16126T>C mutation group, all of whom died within 12 months. In contrast, the D-loop wild-type group, comprising 18 glioblastoma patients, reached median survival probability after about 18 months.

#### 2.1.4. Regression Analysis

In the next step, we performed a backward stepwise linear regression to identify factors significantly affecting overall survival. The analysis included the following variables: age, sex, Ki67 expression, ATRX status, p53 IHC mutations, and the D-loop m.16126T>C variant. It revealed that, in addition to the intercept (*p* < 0.001), both the D-loop m.16126T>C variant (*p* = 0.010) and p53 status (*p* = 0.003) notably influenced the survival duration.

### 2.2. Discussion

The sequencing of the human mtDNA revealed a compact organization with minimal noncoding bases between genes, often relying on post-transcriptional polyadenylation for termination codon creation [25]. Although the role of mtDNA mutations in brain tumor genesis remains to be fully elucidated, our study focused on a significant site that could potentially impact prognosis. While this finding may not directly influence therapeutic approaches, it underscores the importance of further exploration into sequencing the mitochondrial tumor genome, which holds promise for advancing liquid biopsy techniques, minimally invasive diagnostics, and disease progression monitoring in the future.

There are few reports on the role of mtDNA mutations in brain tumors (Table 2). Kirches et al. (1999) investigated D-loop heteroplasmy in astrocytic brain tumors, revealing a low degree of heteroplasmy in glioblastoma. They also identified mtDNA mutations in glioblastoma, observing new length variants and instability in repeat regions, suggesting enhanced somatic mutations in gliomas [12]. Mohamed Yusoff et al. (2017) provided evidence of mtDNA D-loop mutations in brain tumors, highlighting the potential of such changes as diagnostic biomarkers [17]. Other authors demonstrated a range of mtDNA variants predisposing to brain tumor development [13]. Interestingly, changes in the length of microsatellite repeats in the D-loop region of the genome were also documented in patients with brain tumors [19]. Our study, together with the above, underscores the complex interplay between mtDNA changes and the genesis of brain tumors, providing insights into their diagnostic and prognostic significance, as well as their potential as therapeutic targets.

Research on the impact of mitochondrial DNA on glioma aggressiveness shows diverse results. Montanini et al. (2005) demonstrated mutations in the D-loop region of mtDNA in 36% of 42 malignant gliomas, yet they did not confirm increased aggressiveness for these tumors, indicating limited utility in prognostic assessment [15]. However, this contradicts other reports as well as our own work. Reverse results were presented by researchers in a recent study, showing the influence of D-loop variants on glioma progression [18]. Our study confirms that mtDNA mutation may affect glioma biology, thus supporting further research in this direction.

The study has a small sample size and is observational, which limits the ability to establish causality. However, the obtained data suggest a potential link between the D-loop m.16126T>C variant and a shorter overall survival time in glioblastoma patients. The observation requires further validation in a multi-center prospective study encompassing a larger study group. Additionally, the significance of this variant should be verified in other CNS tumors.

The establishment of MITOMAP as a comprehensive database for human mitochondrial DNA serves as a resource and a baseline for mitochondrial genome analysis [26]. The m.16126T>C mutation of mtDNA, located in the control region (CR) at positions HVS1/HV1 and TAS2, has been observed with a frequency of 11.820% in Mitomap GB data, 14.321% in gnomAD v3.1 data, and 17.653% in Helix data, which may be explained by its location in the hypervariable region (HV-I) [22]. This mutation meets the criteria for BA1 classification, indicating a benign variant with a frequency of over 1%. It has been classified as benign by both ClinGen and Mitomap, suggesting it is unlikely to cause clinical significance or pathology.

There are a few potential explanations for the failure of variant detection in the material derived from a liquid biopsy. First of all, the blood-brain barrier can limit the release of both cells and tumor-derived DNA into the bloodstream [27]. Furthermore, current methodologies for liquid biopsy may lack the sensitivity and specificity required to detect glioblastoma markers at low abundance. To minimize this risk, we decided to use the ddPCR approach, facilitating a molecular assessment with a small amount of biological material [28]. It is worthwhile to emphasize that even when a significant amount of high-quality tumor-derived DNA can be isolated, the detection of variants may be challenging due to intratumoral heterogeneity [29,30]. Finally, understanding the clinical relevance of the detected markers may be highly challenging and could require further extensive studies.

Our experience with liquid biopsy for non-invasive prognostic marker identification in glioblastoma has highlighted the challenge of obtaining a reliable quantity of DNA for further molecular analysis. With significant interest and adequate funding in this research area, advancements in the isolation and analysis of tumor biological materials are anticipated. These improvements are expected to facilitate the broader application of liquid biopsy in diagnosing CNS tumors and monitoring disease progression.

### 2.3. Strengths and Limitations

The fifth edition of the WHO Classification of CNS tumors encompasses a wide range of brain tumors, including various types of gliomas such as adult-type diffuse glioma, low-grade and high-grade pediatric-type diffuse glioma, and circumscribed astrocytic gliomas, each significantly differing from one another. In our study, we focus on the most aggressive entity—glioblastoma. This approach facilitated a more reliable statistical comparison of the obtained data.

The study’s primary limitation lies in its small sample size. Moreover, being observational, it cannot establish causality between mtDNA D-loop mutations and clinical outcomes. The analysis exclusively focused on the mtDNA D-loop mutation m.16126T>C and overlooked other potential mutations or genetic markers impacting glioblastoma progression. Additionally, due to the urgency of the initial investigation, long-term clinical outcome data are absent.

### 2.4. Conclusions

Our study underscores the significance of examining mtDNA D-loop mutations in glioblastoma patients. Although genetic changes have been known to play a vital role in glioma advancement, the precise contribution of mtDNA mutations is still not fully understood. Mutations in the mtDNA D-loop, like m.16126T>C identified in tumor samples, could potentially influence survival outcomes. These results motivate additional investigation into mtDNA mutations as possible biomarkers for tailoring therapy and predicting prognosis in glioblastoma patients.

## 3. Materials and Methods

In adherence to the principles outlined in the Declaration of Helsinki, a prospective case-control study was conducted with approval from the Institutional Review Board at the Medical University of Lodz, Poland (approval number RNN/139/22/KE). Subjects were enrolled in the study only after meeting all inclusion criteria and providing informed written consent.

### 3.1. Patients

The research cohort comprised 22 Caucasian individuals (100% Poles) undergoing surgical removal of glioblastoma at the Department of Neurosurgery, Medical University of Lodz in Poland between June 2022 and December 2022. For comparison, the control cohort included 25 sex- and age-matched volunteers who underwent surgery for degenerative disc disease or carpal tunnel syndrome. Criteria for exclusion in both groups encompassed severe systemic illness, malnutrition, altered consciousness, corticosteroid treatment, and concurrent malignancies. Additionally, patients with brainstem and infratentorial lesions were excluded from the study.

### 3.2. Routine Immunohistochemistry Staging

The histopathological diagnosis was established by two neuropathologists (MB, KJ). Immunohistochemical staining was conducted on 5 μm thick formalin-fixed paraffin-embedded tissue samples. The antibodies used included rabbit monoclonal anti-epidermal growth factor receptor (EGFR) antibody (Abcam, Cambridge, UK, ab40815, diluted 1:300), rabbit polyclonal anti-ATRX antibody (Abcam, Cambridge, UK, ab97508, diluted 1:300), mouse monoclonal anti-IDH1 R132H (Dianova, Hamburg, Germany, DIA-H09, RTU), monoclonal mouse anti-p53 antibody (Dako, Glostrup, Denmark, Clone D0-7), monoclonal mouse anti-Ki-67 (Dako, Glostrup, Denmark, clone MIB-1), and polyclonal rabbit anti-glial fibrillary acidic protein (GFAP) antibody (Dako, Glostrup, Denmark,). The anti-IDH1 R132H antibody (Dianova, Hamburg, Germany, DIA-H09, RTU) was chosen for its ability to detect the most common IDH1 mutation via immunohistochemistry. Immunohistochemical analysis was performed using an automatic system (AutostainerLink 48, Dako, Glostrup, Denmark,) following standard protocols. EGFR, ATRX, p53, and GFAP staining were categorized as positive/high expression (more than 50% of tumor cells showing expression) or negative/low expression (none or less than 50% of tumor cells showing expression). IDH1 R132H staining was considered positive only if strong cytoplasmic staining was observed in the majority of tumor cells. The Ki-67 index represented the percentage of immunoreactive tumor cell nuclei among the total cell count, with the exclusion of necrotic areas and vascular endothelium from the analysis.

### 3.3. Material Preparation and DNA Isolation

Formalin-fixed paraffin-embedded (FFPE) glioblastoma samples and patients’ plasma obtained before surgery were used in our study. Genomic DNA was isolated using the Tissue DNA Purification Kit (EURX, Gdansk, Poland).

FFPE material was firstly deparaffinized using xylene and double-washed with 96% ethanol. Then LyseT lysis buffer was added. RNaseA and Proteinase K (2 µL and 20 µL, respectively) were added, and the samples were incubated at 56 °C for 1 h. The prepared samples were then subjected to isolation according to the manufacturer’s protocol (Tissue DNA Purification Kit; EURX Gdansk, Poland), utilizing SolT reagent and WashTX1 and WashTX2 buffers. Finally, 50 µL of eluate was obtained.

Fasting peripheral blood samples were collected into tubes containing ethylenediaminetetraacetic acid (EDTA) before the surgical procedure as part of routine preoperative examination. Cells were separated from plasma by centrifugation for 10 min at 7000× *g*. Subsequently, the samples were stored in a refrigerator at −80 degrees Celsius. The total duration of this phase was limited to 30 min. DNA isolation from plasma was performed using 200 µL of plasma. The first step involved adding RNaseA (2 µL) and incubating at room temperature for 5 min. Proteinase K (10 µL) was then added to the samples. Subsequent steps, using SolT buffer, Wash TX1, and Wash TX2, proceeded for samples from paraffin blocks. A 50 μL eluate was obtained. Quantitative DNA analysis for samples isolated from paraffin blocks was performed using the Picodrop spectrophotometer.

### 3.4. Mutation Identification

The mutation analysis of mtDNA D-loop m.16126T>C was conducted using the droplet digital PCR (ddPCR) method. Probes and primers were designed using the Primer3 and verified with NetPrimer. They were further optimized in terms of: primer annealing temperatures and dilutions. Additionally, positive controls containing the studied mutation were synthesized.

DdPCR was performed in a multiplex format using 11 μL of ddPCR Supermix for Probes (no dUTP, Bio-Rad, Hercules, CA, USA), 1.1 μL of ddPCR Mutant Assay HEX (Bio-Rad), 1.1 μL of ddPCR Wild-type Assay FAM, and 8 μL (maximum volume) of DNA isolated from serum. The amount of DNA from paraffin blocks was 0.008 ng. Water was used as a negative control (NTC). Droplets were generated using the QX 200 droplet generator (Bio-Rad, Hercules, CA, USA) in an 8-well plate containing 20 μL of reaction mixture and 70 μL of oil. 40 μL of the prepared mixture was transferred to a 96-well plate. The PCR reaction was performed in the T100 Thermal Cycler (Bio-Rad, Hercules, CA, USA) in the following thermal profile: initial denaturation at 95 °C for 10 min, followed by 40 cycles of denaturation at 94 °C for 30 s, annealing at 65 °C for 15 s, and extension at 98 °C for 10 min. The final step involved holding the reaction at 4 °C indefinitely. The temperature ramp rate was set at 2 °C/s. The fluorescence signal intensity was measured using the QX 200 droplet reader (Bio-Rad, Hercules, CA, USA). Samples with a droplet count greater than 10,000 were selected for analysis.

### 3.5. Primer Design Strategy

**Figure 2:**
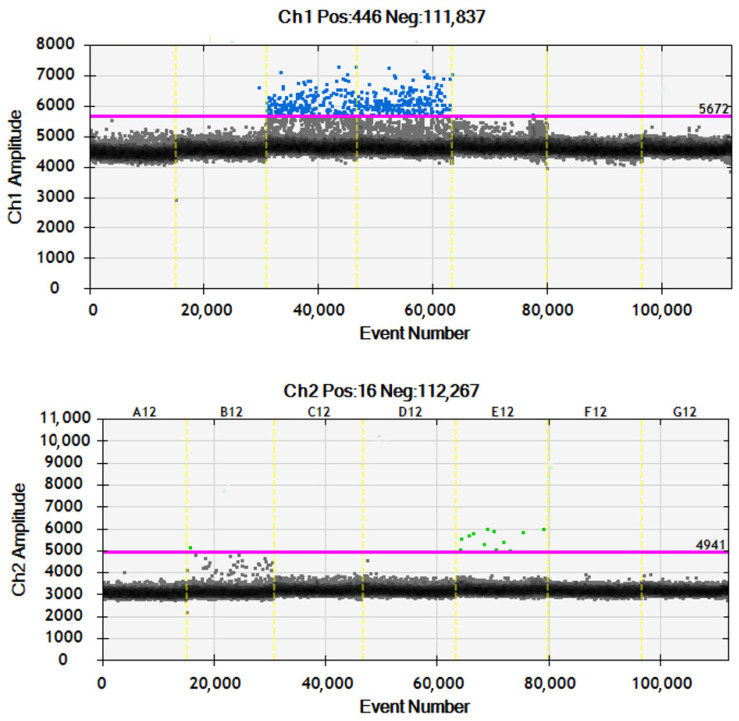
The figure illustrates the analysis of the D-loop region across different sample types: plasma (columns A12 and B12), formalin-fixed paraffin-embedded samples (columns C12 and D12), mutant samples (column E12), and negative controls (NTC) (columns F12 and G12). Each column represents a specific sample type, allowing for the comparison of amplification results across different experimental conditions.

We designed left and right primers along with an internal oligo for the amplification of the mtDNA D-Loop region, specifically targeting both wild-type (WT) and mutant (MUT) sequences. Primer sequences were designed to match the respective regions of interest: F: TCAACAACCGCTATGTATTTCGT, R: GGGTTTTGATGTGGATTGGGT. Internal oligo sequences were employed, with the wild-type internal oligo sequence being CCAGCCACCATGAATATTGTACG and the mutant internal oligo sequence being CCAGCCACCATGAATATTGCACG. The resulting amplicon size for both wild-type and mutant sequences was determined to be 112 base pairs (bp). This design strategy allowed for the precise discrimination between wild-type and mutant sequences within the mtDNA D-Loop region, essential for accurate mutation identification and analysis in subsequent experiments (see Figure 2).

### 3.6. Statistical Analyses

The evaluation of the normality for the distribution of continuous data was conducted using the Shapiro–Wilk test. Data with normal distribution were represented as means with standard deviations and analyzed using parametric tests. Otherwise, medians with interquartile ranges with nonparametric tests were applied. For nominal data, the presentation was in the form of n (% of total). Given the limited size of the smallest subgroup, Fisher’s exact test was chosen for further assessments. A *p*-value of less than 5% was deemed statistically significant. All statistical analyses were executed using Statistica software version 13.1PL (StatSoft, Inc., Tulsa, OK, USA). Additionally, survival analysis, incorporating Kaplan–Meier curves and the log-rank test, was conducted using GraphPad Prism Version 7.0 (GraphPad Software, San Diego, CA, USA).

### 3.7. Literature Search

We employed the following search query for the PubMed database: “D-loop” AND (“glioma” OR “glioblastoma” OR “brain tumor”) on 16-03-2024. The search strategy yielded only 11 papers, each of which underwent a meticulous double-checking process. Subsequently, all identified original papers were incorporated into the further review.

## Figures and Tables

**Table 1:** Demographic and clinical characteristics of study and control groups.

Characteristic	Study Group (n = 22)	Control Group (n = 25)
Age (median, IQR)	64 (45–72)	58 (48–73)
Sex (female/male)	12/10	12/13
*DD-PCR*		
D-loop Mut. Tumor (%)	4 (18%)	N/A
D-loop Mut. Plasma (%)	0	0
*Immunohistochemistry*		
IDH1 Mutation (%)	2 (9%)	N/A
GFAP Expression (%)	22 (100%)	N/A
Ki67 (median, IQR)	30% (19–40)	N/A
ATRX Loss (%)	11 (50%)	N/A
P53 Expression (%)	6 (27%)	N/A
12-month mortality (%)	9 (41%)	N/A
Mortality (%)	12 (55%)	N/A
Survival (median, IQR)	9 months (6–14)	N/A
Follow-up (median, IQR)	14 months (9–16) ^1^	N/A

^1^ At the time of publication, ten patients remained on post-radiotherapy temozolomide treatment. Legend: DD-PCR—droplet digital polymerase chain reaction, N/A—not applicable.

**Table 2:** Chronological summary of findings from studies on mtDNA D-loop mutations in gliomas.

No.	Findings	Reference
1	Loss of D-loop heteroplasmy in glioblastoma.	[11]
2	The analysis of gliomas and corresponding blood samples revealed instability in mtDNA D-loop repeat regions and identified somatic mutations, suggesting an amplification of mechanisms generating mtDNA polymorphisms in gliomas.	[12]
3	Most of the mtDNA variants in the D-loop region found in tumors are neutral regarding mitochondrial function and unlikely to impact tumor formations.	[14]
4	Despite the high frequency of mtDNA mutations in gliomas, they do not directly contribute to increased aggressiveness, potentially due to clonal evolution driven by mutations in nuclear genes that promote rapid growth and infiltration of surrounding tissues. However, they may have diagnostic value as genetic markers for tumors.	[15]
5	The D-loop region exhibited the highest mutation frequency among 12 glioblastoma cell lines.	[16]
6	Description of the complete mitochondrial genome sequence of a mouse inbred model.	[21]
7	Half of the brain tumor patients carried somatic D-loop mtDNA mutations, mostly homoplasmic. Mutations were more common in men and in patients older than 45 years.	[17]
8	The study revealed an association between mtDNA variants of the D-loop, such as C16069T, T16126C, C16186T, G16274A, C16355T, and T16362C, and brain tumor risk, highlighting the potential role of these variations in the pathogenesis of brain tumors.	[13]
9	D-loop mtDNA variants C16223T, T16189C, T16311C, and T16126C in gliomas were found to be relevant to WHO classification and morphological grade, highlighting their potential as tumor biomarkers for assessing tumor risk and progression.	[18]
10	A relatively high prevalence of microsatellite instability (mtMSI) in the D-loop region of mitochondrial DNA (mtDNA) has been demonstrated in brain tumors, with no significant association found with clinical features.	[19]
11	tRNA-derived small RNAs originating from D-loop and T-loop mtDNA fragments were identified in glioma samples. Some of these tsRNAs were found to be down-regulated in gliomas and correlated with poorer survival outcomes, while others demonstrated the potential to inhibit glioma cell proliferation and tumor growth in vivo. Certain tsRNAs may play a role in glioma progression.	[20]

## Data Availability

All data are included in the article.

## References

[1] Louis D.N., Aldape K., Brat D.J., Capper D., Ellison D.W., Hawkins C., Paulus W., Perry A., Reifenberger G., Figarella-Branger D. (2017). Announcing CIMPACT-NOW: The Consortium to Inform Molecular and Practical Approaches to CNS Tumor Taxonomy. Acta Neuropathol..

[2] Ebner V., Gupta P., Vibhute A.K., Agarwal R.K., Rigsby P., Brahmbhatt A.B., Desai G., Bathla B.A., Rigsby R.K., Brahmbhatt P. (2023). Newly Recognized CNS Tumors in the 2021 World Health Organization Classification: Imaging Overview with Histopathologic and Genetic Correlation. Am. J. Neuroradiol..

[3] Poon M.T.C., Sudlow C.L.M., Figueroa J.D., Brennan P.M. (2020). Longer-Term (≥2 Years) Survival in Patients with Glioblastoma in Population-Based Studies Pre- and Post-2005: A Systematic Review and Meta-Analysis. Sci. Rep..

[4] Bigner S.H., Mark J., Burger P.C., Mahaley M.S., Bullard D.E., Muhlbaier L.H., Bigner D.D. (1988). Specific Chromosomal Abnormalities in Malignant Human Gliomas. Cancer Res..

[5] Stoczynska-Fidelus E., Szybka M., Piaskowski S., Bienkowski M., Hulas-Bigoszewska K., Banaszczyk M., Zawlik I., Jesionek-Kupnicka D., Kordek R., Liberski P.P. (2011). Limited Importance of the Dominant-Negative Effect of TP53missense Mutations. BMC Cancer.

[6] Kozakiewicz P., Grzybowska-Szatkowska L., Ciesielka M., Całka P., Osuchowski J., Szmygin P., Jarosz B., Ostrowska-Leśko M., Dudka J., Tkaczyk-Wlizło A. (2023). Mitochondrial DNA Changes in Respiratory Complex I Genes in Brain Gliomas. Biomedicines.

[7] Leão Barros M.B., Pinheiro D.D.R., Borges B.D.N. (2021). Mitochondrial DNA Alterations in Glioblastoma (GBM). Int. J. Mol. Sci..

[8] Su J., Li Y., Liu Q., Peng G., Qin C., Li Y. (2022). Identification of SSBP1 as a Ferroptosis-Related Biomarker of Glioblastoma Based on a Novel Mitochondria-Related Gene Risk Model and in Vitro Experiments. J. Transl. Med..

[9] Kasamatsu H., Robberson D.L., Vinograd J. (1971). A Novel Closed-Circular Mitochondrial DNA with Properties of a Replicating Intermediate. Proc. Natl. Acad. Sci. USA.

[10] McIlwraith M.J., West S.C. (2008). DNA Repair Synthesis Facilitates RAD52-Mediated Second-End Capture during DSB Repair. Mol. Cell.

[11] Kirches E., Michael M., Woy C., Schneider T., Warich-Kirches M., Schneider-Stock R., Winkler K., Wittig H., Dietzmann K. (1999). Loss of Heteroplasmy in the Displacement Loop of Brain Mitochondrial DNA in Astrocytic Tumors. Genes Chromosom. Cancer.

[12] Kirches E., Krause G., Warich-Kirches M., Weis S., Schneider T., Meyer-Puttlitz B., Mawrin C., Dietzmann K. (2001). High Frequency of Mitochondrial DNA Mutations in Glioblastoma Multiforme Identified by Direct Sequence Comparison to Blood Samples. Int. J. Cancer.

[13] Altafi D., Sadeghi S., Hojatian H., Afra M.T., Kar S.P., Gorji M., Houshmand M. (2019). Mitochondrial Polymorphisms, in the D-Loop Area, Are Associated with Brain Tumors. Cell J..

[14] Vega A., Salas A., Gamborino E., Sobrido M.J., Macaulay V., Carracedo Á. (2004). MtDNA Mutations in Tumors of the Central Nervous System Reflect the Neutral Evolution of MtDNA in Populations. Oncogene.

[15] Montanini L., Regna-Gladin C., Eoli M., Albarosa R., Carrara F., Zeviani M., Bruzzone M.G., Broggi G., Boiardi A., Finocchiaro G. (2005). Instability of Mitochondrial DNA and MRI and Clinical Correlations in Malignant Gliomas. J. Neurooncol..

[16] Yeung K.Y., Dickinson A., Donoghue J.F., Polekhina G., White S.J., Grammatopoulos D.K., McKenzie M., Johns T.G., John J.C.S. (2014). The Identification of Mitochondrial DNA Variants in Glioblastoma Multiforme. Acta Neuropathol. Commun..

[17] Yusoff A.A.M., Nasir K.N.M., Haris K., Khair S.Z.N.M., Ghani A.R.I.A., Idris Z., Abdullah J.M. (2017). Detection of Somatic Mutations in the Mitochondrial DNA Control Region D-loop in Brain Tumors: The First Report in Malaysian Patients. Oncol. Lett..

[18] Kilicturgay Yuksel S., Ozduman K., Yilmaz E., Pamir N., Boylu Akyerli C. (2020). Analysis of Mitochondrial Dna Control Region D-Loop in Gliomas: Result of 52 Patients. Turk. Neurosurg..

[19] Abd Radzak S.M., Mohd Khair S.Z.N., Ahmad F., Idris Z., Mohamed Yusoff A.A. (2020). Accumulation of Mitochondrial Dna Microsatellite Instability in Malaysian Patients with Primary Central Nervous System Tumors. Turk. Neurosurg..

[20] Ren J., Wu X., Shang F.-F., Qi Y., Tang Z., Wen C., Cao W., Cheng Q., Tan L., Chen H. (2022). The TRNA-Cys-GCA Derived TsRNAs Suppress Tumor Progression of Gliomas via Regulating VAV2. Dis. Markers.

[21] Gao H.-Z., Wang M.-X., Hu W.-P., Chen J.-Y., Chen X.-R., Lin L. (2014). Complete Mitochondrial Genome Sequence and Mutations of the Glioma Model Inbred C57BL/6 Mice Strain. Mitochondrial DNA.

[22] Sharawat S.K., Bakhshi R., Vishnubhatla S., Bakhshi S. (2010). Mitochondrial D-loop Variations in Paediatric Acute Myeloid Leukaemia: A Potential Prognostic Marker. Br. J. Haematol..

[23] Soffietti R., Bettegowda C., Mellinghoff I.K., Warren K.E., Ahluwalia M.S., De Groot J.F., Galanis E., Gilbert M.R., Jaeckle K.A., Le Rhun E. (2022). Liquid Biopsy in Gliomas: A RANO Review and Proposals for Clinical Applications. Neuro. Oncol..

[24] Bobeff E.J., Szczesna D., Bieńkowski M., Janczar K., Chmielewska-Kassassir M., Wiśniewski K., Papierz W., Wozniak L.A., Jaskólski D.J. (2021). Plasma Amino Acids Indicate Glioblastoma with ATRX Loss. Amino Acids.

[25] Anderson S., Bankier A.T., Barrell B.G., de Bruijn M.H.L., Coulson A.R., Drouin J., Eperon I.C., Nierlich D.P., Roe B.A., Sanger F. (1981). Sequence and Organization of the Human Mitochondrial Genome. Nature.

[26] Kogelnik A.M., Lott M.T., Brown M.D., Navathe S.B., Wallace D.C. (1996). MITOMAP: A Human Mitochondrial Genome Database. Nucleic Acids Res..

[27] Ronvaux L., Riva M., Coosemans A., Herzog M., Rommelaere G., Donis N., D’Hondt L., Douxfils J. (2022). Liquid Biopsy in Glioblastoma. Cancers.

[28] Izquierdo E., Proszek P., Pericoli G., Temelso S., Clarke M., Carvalho D.M., MacKay A., Marshall L.V., Carceller F., Hargrave D. (2021). Droplet Digital PCR-Based Detection of Circulating Tumor DNA from Pediatric High Grade and Diffuse Midline Glioma Patients. Neuro-Oncol. Adv..

[29] Klekner Á., Szivos L., Virga J., Árkosy P., Bognár L., Birkó Z., Nagy B. (2019). Significance of Liquid Biopsy in Glioblastoma—A Review. J. Biotechnol..

[30] Auzmendi-iriarte J., Carrasco-garcia E., Moreno-cugnon L., Ruiz I., Villanua J., Otaegui D., Matheu A. (2019). Liquid Biopsy in Glioblastoma: Opportunities, Applications and Challenges. Cancers.

